# Persuasive design features within a consumer-focused eHealth intervention integrated with the electronic health record: A mixed methods study of effectiveness and acceptability

**DOI:** 10.1371/journal.pone.0218447

**Published:** 2019-06-20

**Authors:** Genevieve Coorey, David Peiris, Tim Usherwood, Lis Neubeck, John Mulley, Julie Redfern

**Affiliations:** 1 The University of Sydney, Faculty of Medicine and Health, Sydney School of Public Health, Sydney, New South Wales, Australia; 2 The George Institute for Global Health, Sydney, New South Wales, Australia; 3 The University of New South Wales, Faculty of Medicine, Sydney, New South Wales, Australia; 4 The University of Sydney, Faculty of Medicine and Health, Department of General Practice, Sydney, New South Wales, Australia; 5 Edinburgh Napier University, School of Health and Social Care, Edinburgh, United Kingdom; Robert Gordon University, UNITED KINGDOM

## Abstract

**Introduction:**

eHealth strategies targeting health-related behaviour often incorporate persuasive software design. To further engage patients with their overall health management, consumer-facing web portals may be integrated with data from one or more care providers. This study aimed to explore effectiveness for healthier behaviour of persuasive design characteristics within a web application integrated with the primary health care electronic record; also patient and general practitioner (GP) preferences for future integrated records.

**Methods:**

Mixed methods study within the Consumer Navigation of Electronic Cardiovascular Tools randomised controlled trial. Participants were patients with moderate-high risk of cardiovascular disease, and their GPs. Survey and web analytic data were analysed with descriptive statistics. Interview and focus group transcripts were recorded, transcribed, coded and analysed for themes.

**Results:**

Surveys (n = 397) received from patients indicated improved medication adherence (31.8%); improved mental health and well-being (40%); higher physical activity (47%); and healthier eating (61%). Users of the interactive features reported benefiting from personalised cardiovascular disease risk score (73%); goal tracking (69%); risk factor self-monitoring (52%) and receipt of motivational health tips (54%). Focus group and interview participants (n = 55) described customisations that would increase portal appeal and relevance, including more provider interaction. Of the GP survey respondents (n = 38), 74% reported increased patient attendance and engagement with their care. For future integrated portals, 94% of GPs were in favour and key themes among interviewees (n = 17) related to design optimisation, impact on workflow and data security.

**Conclusion:**

Intervention features reflecting the persuasive design categories of Primary Task support, Dialogue support and System Credibility support facilitated healthier lifestyle behaviour. Patients valued customisable functions and greater patient-provider interactivity. GPs identified system challenges but saw advantages for patients and the health care relationship. Future studies could further elucidate the persuasive design principles that are at play and which may promote adoption of EHR-integrated consumer portals.

## Introduction

Digital health interventions are increasingly being used to assist patients with or at risk of chronic conditions to adopt and sustain lifestyle changes. Functions of such interventions include the ability to track and record personal biometric data, provide disease-specific information or instructions, promote desired lifestyle choices, and for medication reminders. Although the evidence base is evolving, they have potential to offer cost, accessibility, convenience and scalability advantages.[1] In the area of cardiovascular disease (CVD) prevention and management, various digital health strategies (including stand-alone software tools, web-based applications and mobile phone-based strategies) have been tested and there is similarly emerging evidence of their ability to improve risk awareness, self-care and uptake of recommendations for dietary behaviour, physical activity levels, smoking cessation, and medication adherence. [2–6]

To date, most consumer-focussed digital health interventions that have been studied are stand-alone applications. Integration with a provider electronic health record (EHR) is a newer area that potentially provides greater opportunities for consumers to engage more actively in their health care. Personal health records, wherein entry and storage of health information is controlled by the consumer, have evolved from paper- or electronic-based tools to Internet-enabled systems supporting more comprehensive functions and interactivity. Tethered health records, also known as patient portals, are connected via a web portal to an institution- or provider-specific EHR.[7–9] Although some tethered models allow patients to enter data into the record [8] and support communication with the clinician,[9] it is foremost a provider-controlled record.[7] Conversely, untethered or integrated health records are maintained by the consumer who can both enter health information and permit data transfer into the record by multiple sources, for example a pharmacy, laboratory or medical service.[7, 8] Overall, these systems go beyond the capabilities of stand-alone personal records to enable access to provider-held data, personal health data entry and self-monitoring, and transactional tasks such as appointments, reminders, prescription renewals and patient-provider email communication. [7]

Among the purported benefits of tethered patient portals and integrated personal health records is improvement in care quality through greater consumer participation in health decision-making; fostering a notion of shared care or a care partnership. [7, 9, 10] Evidence is building of consumer interest and increased care participation and satisfaction with such systems.[11–14] Internationally, the scale of tethered record implementation varies from, for example, regional clusters of primary health care providers [9] to large-scale organisations. [14, 15] Various country-specific initiatives for a national-level personal health data repository differ by functionality offered, including EHR interactivity. [16] Implementation models that require individual providers to introduce personal health records to patients tend to be poorly subscribed; [17] whereas a nationally-deployed initiative with low voluntary uptake has been disbanded. [18] In the Australian health care system, consumer and provider experience with models of either tethered or integrated health record innovations is limited. Currently, a national scheme of personally-controlled electronic health records is being implemented. Providers from various locations can upload information about a health encounter into the centralised record, with the patient’s permission; also, the patient can enter or update information within some sections of the record. A key purpose is to facilitate timely provision of essential health information to care providers to whom the consumer permits access to the record. [10] As with an integrated health record model, the record is managed chiefly by the consumer.

In this paper we outline the process evaluation of a multi-feature consumer-facing web application that was integrated with selected parts of the primary health care EHR and tested in a randomised controlled trial (RCT). It was hypothesised that this application would improve CVD risk factor control for people with or at high risk of CVD through improved health-related behaviours, including engagement with primary health care providers, healthier lifestyle, and better medication adherence. [19] Further, integration with data from the EHR was expected to strengthen the intervention’s effect on patients’ attitude and behaviour towards CVD risk factor improvement. The specific aims of the process evaluation were to (1) identify the triggers and motivators for healthier behaviour within an intervention employing persuasive software characteristics and integration with the EHR; (2) explore general practitioner (GP) perspectives on the EHR-integrated consumer portal; and (3) identify the preferences for future approaches to health record integration from the perspectives of patients and GPs.

## Methods

### Design

The protocols for the RCT and this process evaluation are reported elsewhere. [19, 20] Participants allocated to the intervention arm had access to the integrated web application in addition to usual health care from their GP; participants allocated to the control arm received usual health care from their GP without access. The follow-up period was 12 months. This evaluation of participant experiences of the intervention was aimed in part at understanding what made it ‘persuasive’, and was by intention conducted blinded to the RCT outcomes, not as an explanation of RCT outcomes. Ethical approval for both the RCT and the process evaluation was received from the Human Research Ethics Committees of the University of Sydney and the New South Wales Aboriginal Health and Medical Research Council.

### Features of the integrated consumer portal

**Fig 1:**
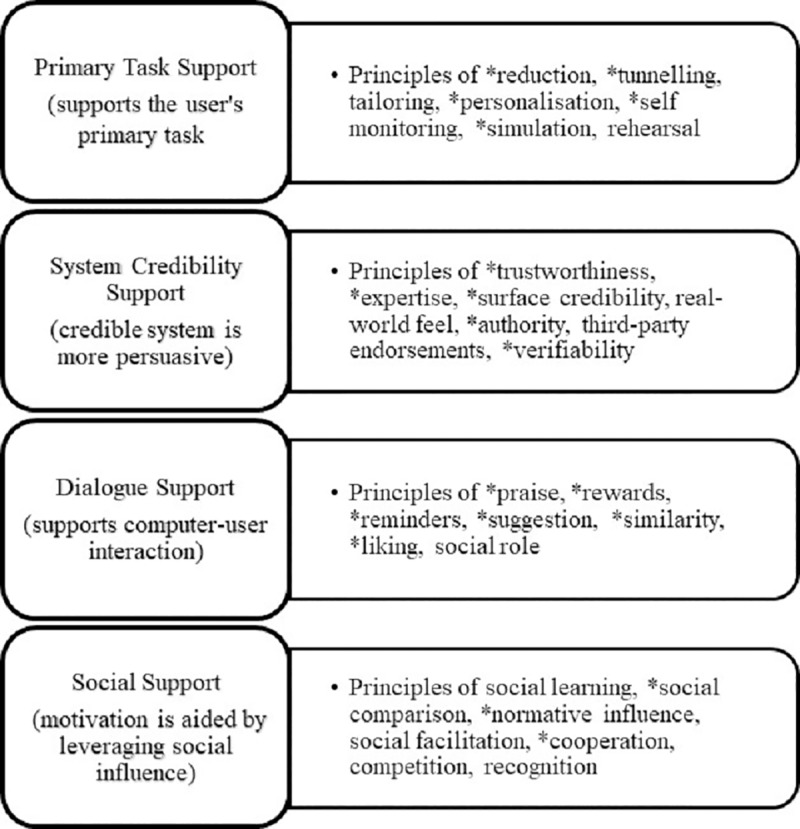
Four categories of persuasive software design with 28 associated category principles. (22) *Principles informing features and functions within the eHealth intervention.

Development of the web application followed a user-centred design approach and has been described elsewhere. [21] Selection of the included features (Box 1) was informed by the four recommended functions of persuasive software, namely Primary Task support (e.g., self-monitoring), Dialogue support (e.g., reminders), System Credibility support (e.g., trustworthiness), and Social support (e.g., social comparison)[22] (Fig 1). Persuasive features are characteristics of a technology that influence the user’s motivation and/or ability to make desired behaviour changes, or provide the trigger(s) for such change, without using coercion or deception.[22, 23] Interactive and personalised features characterising the persuasive intent of the intervention aimed to assist patients to learn about their own CVD risk factor profile, and modify lifestyle behaviours to reduce risk. At study baseline, selected medical data were uploaded into a personalised portal that was securely integrated with the primary health care EHR and accessible on any internet-enabled device. Thereafter, the portal was updated after any medical encounter with the GP in which changes to those data occurred; for example, medical diagnoses, prescribed medications, physical measurements (weight, waist circumference, blood pressure), cholesterol concentration, and for diabetic patients the glycosylated haemoglobin results. The entire EHR content was not visible to the patient, nor were patient entries to the portal transmitted back into the EHR.

**Box 1: Features of the EHR-integrated application**

**1**. **Personalized absolute CVD risk score estimation**

- Based on updateable EHR data, participants saw how their risk factor status affected the score and heart age
- Interactive sliders simulated changed heart age and risk score based on changes to modifiable risk factor values
- Information about risk factor control linked to the goal setting

**2**. **Goal setting, tracking and virtual rewards**

- Participants could set, track and modify self-chosen goals around healthier eating, physical activity, smoking cessation and emotional well-being
- Virtual rewards accumulated for weekly and monthly goal achievement
- Linked also to the self-monitoring and EHR-derived charts of risk factor values

**3**. **Interactivity with EHR-derived information**

- Participants used interactive screens to log measurements and results into the charts alongside EHR-derived updates
- Enabled tracking of progress on key risk factors (e.g., blood pressure, cholesterol)
- Care navigation was further assisted with information specific to each prescription medication and calendar links enabling the patient to see due dates for future tests

**4**. **Social media chat forum/message board**

- Participants could write comments, ask questions or share stories with peers, anonymously if they preferred
- Research staff monitored the content and responded to general questions; participant questions that were of a more personal nature were answered outside the community forum

**5**. **Heart health tips, motivational messages and reminders**

- Semi-personalised content related to healthy lifestyle and medication knowledge and adherence
- Delivered on a programmed frequency via email and/or text message
- Participants could opt into receiving messages via one or both formats and at any time could opt out entirely or temporarily, for example when traveling overseas

Abbreviations: CVD, cardiovascular disease; EHR, electronic health record.

### Data sources

This study drew on qualitative and quantitative data from four sources which have been described previously.[20] The consolidated criteria for reporting qualitative research [24] were used to guide reporting of the focus groups and interviews.

1. **Feedback surveys**. Patients in the intervention arm of the RCT completed a survey after the 12-month follow-up period (S1 File). The aims of the survey were to determine (1) which intervention features were used; (2) whether these features helped patients better understand CVD and risk factors they could modify; (3) the impact on lifestyle, for example weekly physical activity; and (4) overall ease of interacting with the application interface. Surveys were completed in person by participants who attended the month 12 study assessment; those who did not attend in person were mailed a survey with a stamped, addressed return envelope.
  All GPs from practices or clinics taking part in the RCT received a survey via postal mail at completion of 12 months of follow-up by participants enrolled from their site (S1 File). Content of the survey explored current practices with lifestyle behaviour counselling to reduce vascular risk factors, and views on benefits and drawbacks of integrating patient-facing eHealth strategies with the primary care EHR. Surveys were returned in a stamped, addressed return envelope supplied with the survey, or by email. Research staff sent up to two reminder emails or telephone calls to patient and GP survey recipients to ensure return of as many surveys as possible.
2. **Web analytic data**. Usage data about the web application were collected for each intervention participant, commencing on the date of their individual ‘go-live’ session with study staff and continuing through the 12-month follow-up period. Each participant’s system logins as well as visits to selected in-app features (for example, CVD risk score estimation, goal setting and progress tracking, or the chat forum) were logged in a purpose-designed database. The logs enabled researchers to quantify interest in various in-app features and compare these findings with those from self-reported feedback.
3. **Focus group discussions and semi-structured interviews (patients).** Focus groups and interviews were held with a sample of participants from the intervention arm of the RCT who had completed the 12-month follow-up period. Purposive sampling was used to ensure participants varied by such characteristics as CVD risk status (existing CVD or at high risk), education level, sex, and extent of use of the intervention. One researcher contacted participants by telephone to explain the purpose of these activities and requested their involvement. An information sheet was mailed or emailed to the participant and written informed consent was obtained on the day. Focus groups and interviews lasted up to one hour and took place in a variety of locations based on convenience for participants. One researcher (GC) facilitated the focus groups and conducted the interviews, all of which were audio-recorded. A non-participant observer attended the focus groups but only the researcher and patient were present at interviews. There was no prior relationship between facilitator and participants except if they had communicated during routine conduct of the RCT. An open-ended discussion/interview guide targeted key areas of interest (S2 File). After each of the focus groups, debriefing occurred between the facilitator and observers, two of whom were experienced focus group facilitators. Researchers did not carry out repeat interviews or return transcripts to interviewees.
4. **Semi-structured interviews (GPs).** Individual interviews with a sub-group of participating GPs were conducted at completion of 12 months of follow-up by RCT participants enrolled from their practice or clinic. Interviewees were selected purposively based on characteristics such as practice size and location within Sydney and surrounds, representing varying demography of patients. A balance of male and female GPs were approached for interviews. Interviews of up to 30-minutes duration took place at the practice or clinic, or by telephone, as preferred by the GP. Written informed consent was obtained from each interviewee. An open-ended discussion guide was used and interviews were audio-recorded (S3 File). Only the researcher (GC) and GP were present and were not known to each other. Additional field notes were not recorded. Researchers did not carry out repeat interviews or return transcripts to interviewees.

### Data analysis and synthesis

Quantitative data findings were integrated with the qualitative data in the analysis and interpretation of the patients’ and the GPs’ experiences of the intervention during the RCT. Quantitative data, for example, were used to ensure variation in sampling of patients and GPs for the interviews and focus groups. Thereafter, qualitative data findings were analysed in parallel with survey and web analytic data to more fully understand the experiences of the intervention. Descriptive statistics were used to summarize the survey responses and demographic data about participants in surveys, focus groups and interviews. Usage of interactive screens/ app features over 12 months of intervention exposure were reported as mean number of visits to each screen by all users. Median values and interquartile range were included because login frequency and screen visits were expected to be irregular both within and between intervention users.

For the qualitative data, an inductive coding approach of interview and focus group data was taken to identify categories and emergent themes. [25] To do this, text from each individual transcription was initially coded to a series of labels about important and interesting aspects of the data that were relevant to the research question. The numerous labels were grouped to an overarching category, then the categories were further consolidated into themes. Illustrative quotes were selected to add the interviewees’ voices to the results and portray the range of insights offered. Themes from patient and GP interviews were compared and contrasted to understand varied expectations and preferences of the intervention. [26] NVivo was used to aid organisation of the transcript texts and codes (QSR International Pty Ltd Victoria, Australia). The features within the intervention that patients identified as useful for healthier lifestyle behaviour were compared with the four support categories of the PSD framework that the intervention was built around. This was to ascertain the extent to which effective motivators and triggers as identified by patients lay in one or more of Primary Task support, Dialogue support, System Credibility support, and Social support categories. Further, PSD components and the key themes within GP and patient feedback were added to the RCT process evaluation logic model. The original logic model has been detailed previously in terms of setting out the intended inputs, activities and outputs within the change process in an eHealth intervention. [20]

## Results

### Participants

Surveys were distributed to 91% of intervention arm participants (444/486) and of these 397/444 (89%) responded. Demographic characteristics of patients who took part in evaluation activities are summarised in Table 1. Surveys were distributed to 51 GPs and 38 responded (75%). Of these, 55% (n = 21) were male and 79% (n = 30) were from practices with three or more GPs. Of the 17 GP interviewees, 65% (n = 11) were male and 65% (n = 11) were from practices with three or more GPs.

**Table 1:** Baseline characteristics of patient participants.

	Survey	Focus groups	Interviews
	(n = 397)	(n = 19)	(n = 36)
**Age, mean (SD)**	66 (8)	69 (6)	67 (8)
**Male % (n)**	76.1 (302)	89.5 (17)	50 (18)
**Highest completed educational qualification** ^*^ **% (n)**			
School only	28.9 (115)	26.3 (5)	50 (18)
Undergraduate degree	20.9 (83)	5.3 (1)	16.7 (6)
Postgraduate degree or diploma	27.9 (111)	31.6 (6)	16.7 (6)
Technical/vocational qualification	21.6 (86)	36.8 (7)	16.7 (6)
**Employment status** ^**†**^ **% (n)**			
Full-time	22.2 (88)	15.8 (3)	8.3 (3)
Part-time	14.1 (56)	21.1 (4)	19.4 (7)
Retired	60.4 (240)	63.2 (12)	72.2 (26)
**CVD status % (n)**			
Existing CVD	43.1 (171)	31.6 (6)	50 (18)
High risk of CVD	56.9 (226)	68.4 (13)	50 (18)
**eHEALS score** ^*^ **% (n)**			
Total score ≥26	70 (278)	79.0 (15)	72.2 (26)
Total score <26	30 (119)	21.0 (4)	27.8 (10)
Mean score	27.3	28.9	27.7
**Self-reported uptake of new technology products % (n)**			
I am generally the first, or among the first	21.7 (86)	36.8 (7)	19.4 (7)
I am generally in the middle	51.4 (204)	42.1 (8)	50 (18)
I am generally the last, or among the last	26.9 (107)	21.1 (4)	30.6 (11)

SD, standard deviation; CVD, cardiovascular disease; eHEALS, electronic health literacy score

^*^ Response not provided or score unavailable in 0.5% (n = 2) of survey respondents.

^**†**^ Survey respondents: Response not provided in 0.3% (n = 1); response ‘not working- other’ in 3% (n = 12).

### General impressions of the integrated web application

Overall, most patients (72%) felt that using the intervention for 12 months was sufficient to evaluate its utility. Web analytic data showed that on average each participant logged in 18 times over 12 months. These data further indicated that goal tracking/progress was the most visited interactive screen and the chat forum the least visited (Table 2). Most survey respondents (89%) felt that information on the screens was clear but 16% felt it was hard to locate the screens they needed. Assistance from study staff was reported as helpful by 42% of respondents. Motivations to improve heart health were: ‘My GP’s advice’ (53.2%, 198/372 respondents); ‘My blood pressure or weight was high’ (41.7%, 155/372); ‘My blood cholesterol level was high’ (29.9%, 110/372); ‘I have a family history of heart disease’ (27.2%, 101/372); and ‘My heart risk score was high’ (20.7%, 77/372). Most GPs ask their patients to track or record measurements in between office visits (76.3%, 29/38) but fewer than half suggest the patient uses technology, for example an App, to support healthier behaviour (44.7%, 17/38). Interestingly, 86.8% (33/38) responded they would be likely or very likely to recommend to their patients an eHealth strategy; 94.1% (31/34) would be in favour of a future integrated intervention for their patients. Overall, patient respondents reported improved medication adherence (31.8%; 119/374 respondents); doing more to improve mental health and well-being (40%; 150/375); higher weekly physical activity (47%; 175/372); and healthier eating habits (61%; 229/373), as a result of the intervention.

**Table 2:** Views per participant of application features over 12 months.

Characteristics	Intervention Participants (n = 414)
Logins	
Mean (SD)	17.7 (34.48)
Median (Q1; Q3)	7.0 (3.0; 15.0)
min max	1 320
Risk factors and EHR-derived data	
Mean (SD)	6.9 (11.84)
Median (Q1; Q3)	3.0 (1.0; 8.0)
min max	0 108
CVD risk Score	
Mean (SD)	8.2 (10.69)
Median (Q1; Q3)	4.0 (2.0; 11.0)
min max	0 66
Goal setting	
Mean (SD)	11.0 (13.56)
Median (Q1; Q3)	7.0 (3.0; 13.0)
min max	0 101
Goal tracking/progress	
Mean (SD)	31.7 (108.97)
Median (Q1; Q3)	6.0 (2.0; 20.0)
min max	0 1941
Social media/Chat forum	
Mean (SD)	5.1 (11.70)
Median (Q1; Q3)	3.0 (1.0; 5.0)
min max	0 199

Abbreviations: CVD, cardiovascular disease; EHR, electronic health record; max, maximum; min, minimum; Q1, first quartile; Q3, third quartile; SD, standard deviation.

### Influence of persuasive category features on CVD awareness and lifestyle behaviour

**Fig 2:**
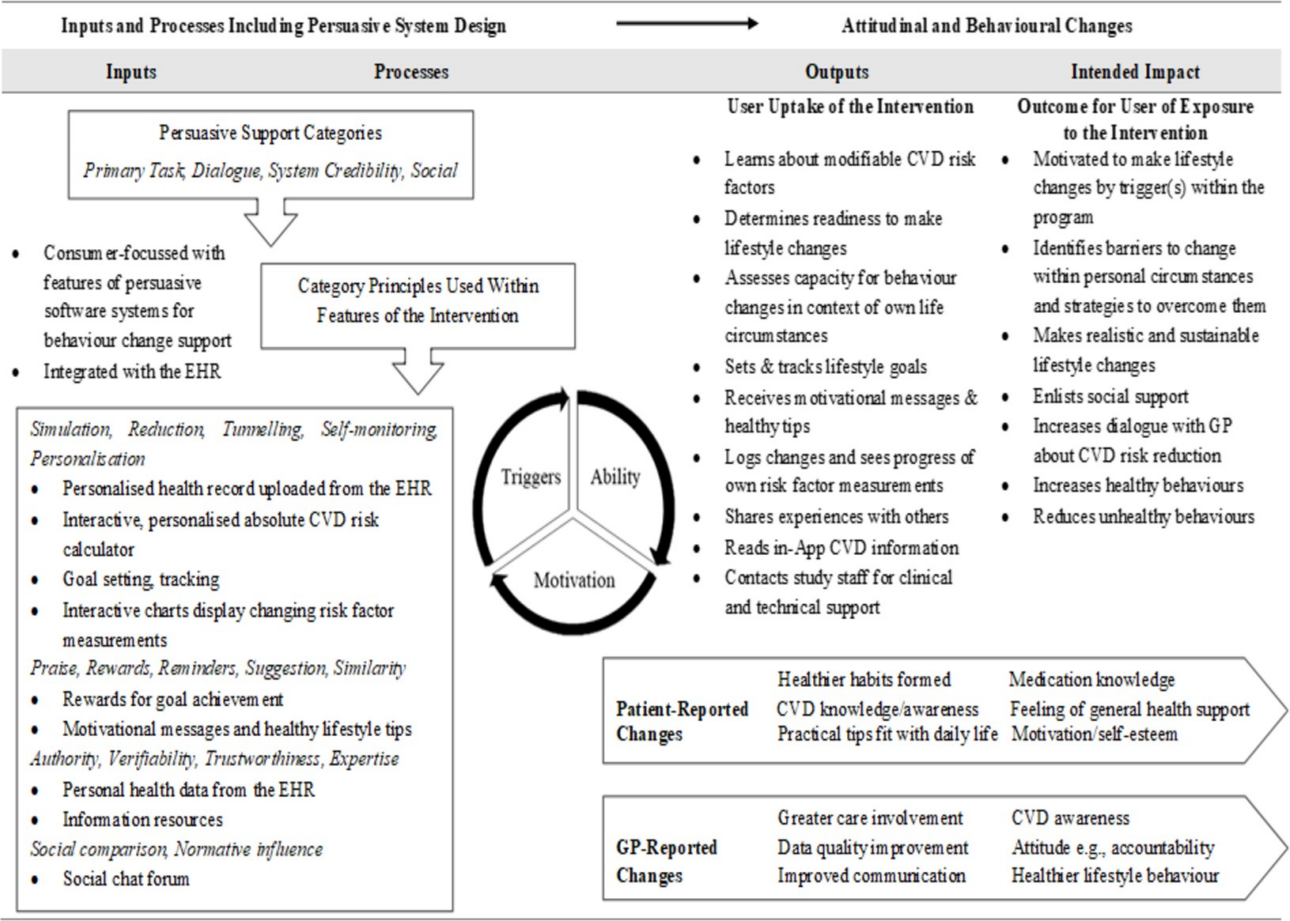
PSD inclusion and change effects within key stages of the RCT logic model Abbreviations: CVD, cardiovascular disease; EHR, electronic health record; GP, general practitioner; PSD, persuasive software design; RCT, randomised controlled trial.

Four persuasive support categories were reviewed in terms of the utility, appeal and effectiveness for facilitating healthier behaviour of the intervention features within them. Emergent themes from participant feedback about five key features are shown in Table 3. In Fig 2, persuasive principles included in the intervention, and important benefits as reported by patients and GPs, are shown in relation to the inputs and outcomes stages of the process evaluation logic model.

**Table 3:** Themes derived from participant feedback about five key features of an integrated eHealth intervention.

Intervention features and applicable persuasion categories	Themes related to intervention utility and appeal
1. Personalised interactive CVD risk score and heart age estimation Primary Task support System Credibility support	Varied visual formats facilitate CVD risk communication Risk score responsiveness to changes in risk factor measurements influences user motivation Health literacy influences perceived credibility of a risk score estimation
2. Healthy lifestyle goal setting and tracking Primary Task support Dialogue support	Healthier choices and actions become habitual rather than occasional Recognition of achievement influences personal motivation and self-esteem Engagement is facilitated by association of behaviour with specific risk factor benefit Goal setting needs to be realistic and personally acceptable
3. Self-monitoring with updateable risk factor and medication input from the EHR Primary Task support System Credibility support	Changes in risk factor values are most successful when they are positive and relatable to everyday behaviour Motivation improves if recent data entries by the patient update the CVD risk factor profile within the EHR Limited choice of data graphs and pre-set risk factor targets can reduce personal relevance of monitoring Personalised prescription medication display from the EHR increases knowledge-seeking
4. Social chat forum/message board Social support	Social and chat forums lack relevance and appeal for those disinterested in social media Sharing common experiences can be helpful to those comfortable with using an anonymous chat forum
5. Semi-personalised, heart healthy lifestyle behaviour tips via email and/or SMS Dialogue support System Credibility support Social support	Receiving tips and reminders conveys a sense of more general health support Receptiveness to the message content is affected by language, frequency and variety Healthier behaviour is positively influenced when message content is delivered repeatedly and perceived to be practical Message receipt benefits new and existing heart health awareness

Abbreviations: CVD, cardiovascular disease; EHR, electronic health record; SMS, short message service.

#### 1. Persuasive category: Primary task support

Features that used principles from this category supported the users’ primary tasks, which in this study were to increase personal CVD risk awareness and adopt or increase healthier behaviours to lower risk, including closer engagement with their primary health care via an EHR-integrated web site. Analytic data showed that intervention features that supported the users’ Primary task had the most views per participant over 12 months (Table 2). Attentiveness to personal health information using technology-based strategies was generally viewed positively by patients. Seventy-nine percent (297/375) of survey respondents reported using the personalised interactive CVD risk score and heart age estimation. Of these, 73% agreed or strongly agreed that the simulation helped them understand how changing risk factors would affect their overall CVD risk.

#### Varied visual formats facilitate CVD risk communication

For some, visual feedback effectively communicated progress or deterioration by reinforcing the feeling of being on track or alerting about potential danger. The ability to simulate modified scores by adjusting data values was a useful attention-grabber regarding the need to improve heart health and for some, prompted a relevant conversation with their GP:

*“I thought it was probably the best part of the study to give people a visual representation…and how that can improve*.*”* (Male interviewee, age 80)

For others, the visual display of risk estimation did not facilitate understanding and other formats would be needed, for example:

*“The heart risk dial—too confronting”*. (Survey respondent)

*“I just find data reading in graphs like that somewhat challenging for me*. *I’m not that visually stimulated*.*”* (Female interviewee, age 61)

#### Risk score changes in response to updated risk factor measurements influenced user motivation

For most respondents, reduction in the risk score over time created a feeling of motivation and positivity; others reported that seeing minimal decrease in their risk score after they improved their physical measurements was demotivating or reduced interest in this metric:

*“My risk has gone down which is quite nice… my age*, *my heart age has gone down…That made me feel good.”(Female interviewee, age 51)*

*“Cholesterol and the heart risk*, *I never even used to think about until I was doing this.” (Male interviewee, age 64)*

*“When I played with the sliders and moved them down to the lowest level my dial only shifted slightly…I thought well that’s not much motivation*. *Maybe if the scale was perhaps a little more sensitive so that the needle moved a bit more, people might think ‘Oh gees, this is worthwhile. I might do that’ but if the needle only goes a couple of millimetres you think ‘Well why bother?’” (Male focus group attendee, age 63)*

Self-monitoring with updateable risk factor and medication input from the EHR was used by 69% (274/397) of participants, of whom 52% (143/274) reported it was helpful to add personal measurements in this way. There were fewer users of the online information resources linked to medications, diagnoses and risk factor data (n = 262) and 39% of these found them to be useful. The integration of self-entered and GP-entered measurements and pathology results was overall well-regarded by participants. Interestingly, it created an expectation both of timely uploads of EHR data into the patient’s portal and of more tailored interpretation of the patient’s data against the CVD guideline-derived targets with which these data were compared. A minority of patients described that if hard copies of pathology results were routinely provided to them, the effort required to log measurements at home did not add interest. For those who did use this feature, changes in risk factor values were most successful when they were positive, and relatable to everyday behaviour: The visual aspect of the charts was more appealing than numbers alone as a way to show trends in physical measurements or blood test results. For some patients, charts showed reward for efforts made to improve a risk factor, or gave a visual indicator of potential problems, and thus were motivating. Participants who were recording measurements at home described how the chart displays prompted reflection about everyday influences on a higher-than–normal measurement (such as blood pressure). Some used the links to further information about a measurement, for example viewing the National Heart Foundation Web site to read more about body mass index.

*“It was a nice way of seeing the graph of the cholesterol thing coming down so that was great–that’s a bonus…a visual feedback on, ‘Oh … maybe I better keep walking, riding, whatever because it’s working’”*. *(Male interviewee, age 55)*

*“I was showing [cardiologist] my pathology data over the last year…he said, ‘What have you been doing*, *this is perfect!’” (Male interviewee, age 65)*

*“I was surprised with some of my blood pressure readings, knowing that what I’d been doing, whether it was the exercise or a combination of the exercise, drinking water and getting rid of the sugars and getting rid of salts and all this sort of thing…I’d go back to ‘well, what’s happened in the last couple of days that perhaps would drive that?’”* *(Male interviewee, age 64)*

#### Motivation improves if recent data entries by the patient update the CVD risk factor profile within the EHR

Participants felt that motivation would have improved if EHR-derived risk factor profile adapted to data entries by the patient. Self-entered measurements did not update the CVD risk score, cholesterol, glycosylated haemoglobin, or physical measurements display derived from the EHR. Patients found this frustrating because their own entries were often more recent than their last clinic visit, were felt to be more accurate, and they had derived motivation from seeing these improvements occurring at home. Many patients mentioned that they would have liked to have their GP see information entered at home and even receive feedback, suggesting future interest in a two-way communication shared health record model.

*“I still get annoyed when I put figures into the graphs and it doesn’t change the risk factors. I got my blood pressure way down*. *I’ve lost weight, and it won’t change.” (Male focus group participant, age 71)*

*“I think in inputting those, you weren’t able to see whether … even though your weight might have come down, whether there was any change to what your risk was…it might be something to make participants be more engaged if they actually saw that if they’re making those changes the impact it had*. *It might have made me feel a bit better.” (Male interviewee, age 61)*

Even among those patients who valued tracking measurements at home, perceived unrealistic targets within the interactive screens, for example body weight, discouraged charting (even if the patient continued efforts to reduce their weight). Similarly, national guideline-recommended targets for total and low-density lipoprotein cholesterol for specific populations, for example those with coronary heart disease, may have differed from the general reference ranges shown on pathology results obtained from a community laboratory. For some participants, such slight differences prompted a conversation with their GP to discuss and agree on a realistic goal.

#### Limited choice of data graphs and pre-set risk factor targets can reduce personal relevance of monitoring

Some participants were disappointed they could not create charts of their own choosing, for example for daily blood glucose level or oral anti-coagulant treatment monitoring, and reported not using the included charts. Thus, the limited choice of data graphs and the pre-set risk factor targets reduced personal relevance even for patients who were interested in self-monitoring:

*“I actually measure all those things all the time, but I never enter them in…because I couldn’t do the [daily glucose level] diabetic ones*. *(Male focus group participant, age 63)*

*“The nominated desirable target limit for cholesterol, HDL, LDL, whatever else–that the software’s giving me that is contrary to for example, the results from the pathology laboratories…I’m within the healthy band for all the cholesterol components but under this app, I’m not*. *(Male interviewee, age 55)*

More generally, participants suggested that a consumer-accessible portal may not hold much appeal for patients who feel well-informed with their health information; who lack interest in their health record outside the face-to-face consultations; who rely on conversations with their GP for information and advice; and who see multiple specialists regularly for a variety of conditions. The latter patients have frequent, regular health conversations and felt that viewing their health encounter information on a web site would be excessive.

Seventy-seven percent (287/372) of respondents reported having set personalised heart health goals in one or more areas of lifestyle. Most participants set goals around healthier eating (84%) and physical activity (82%). Goal-setting was notably lower for well-being/mental health (37.6%) and smoking cessation (11%). In terms of *tracking* goals, 76% of respondents (289/ 379) reported using this feature. Of these, 69% agreed that doing so helped them focus on their heart health. People who found this feature less useful had generally well-established routines for adopting healthy habits and found electronically recording and tracking goals was of minimal interest. There were several effects of using this feature on the primary task of facilitating healthier lifestyle:

#### Healthier choices and actions become habitual rather than occasional

When the healthy behaviour was expressed or described as goals, it positively affected everyday choices and actions. Goal setting and tracking heightened priority of, and attention to, healthy behaviour in daily routine, thereby increasing the chance of it occurring in the day, even if only by increasing incidental physical activity, for example. The regularity of goal tracking helped establish the activity as a habit.

*“Even though you’re thinking, “Oh, I’ll do this, and I’ll do that”, but the fact that I set those goals…I thought, “Well, fish, fruit, salt, sugar” and I sort of ticked them off as I went along*.*” (Female interviewee, age 58)*

*“I did actually increase my activity levels because obviously when you see a full week, “Oh there’s seven days there. I’ve got a gap there. I’d better do something”*. *So that helped; I increased it a little bit.” (Male interviewee, age 55)*

#### Engagement is facilitated by association of behaviour with specific risk factor benefit

Participants described a preference for a mixture of prescribed and personalised self-chosen healthier lifestyle goals rather than relying solely on self-selected goals to be beneficial for their situation. Participants suggested that endorsement and ranking of the goals by the GP may heighten their significance and help people prioritise their efforts:

*“I felt the section where you could put your own goals in was good, because you’re not going to get a one size fits all category, and that’s very important for people to feel that it is tailored to their specific needs*.*” (Male focus group participant, age 63)*

*“One of the things that would be good in terms of ranking the goals would be to say “this is likely to affect this range of this particular aspect of your health”*. *(Male focus group participant, age 66)*

#### Goal setting needs to be realistic and personally acceptable

For many patients, goal setting for health was closely related to an affinity for setting goals in other parts of life. Routinely tracking goals was helpful but the task of logging into an electronic diary was mentioned as a potential barrier over time. Customising was seen as preferable for goal setting and tracking within the program and could take various forms. For example, guideline recommendations for daily intake of fruit and vegetables may not have been immediately achievable but the participant adapted some aspect(s) of their routine towards the target behaviour(s). In this way, better choices were made incrementally. Participants described how setting goals around recommended lifestyle behaviour required concessions for intercurrent health problems, for example musculoskeletal ailments, that could impede their ability to achieve them. Further, participants saw value in being able to record reasons for missed goal achievement within the electronic tracker, so that they could see how other daily events impact their intentions.

*“They can't exist together; exercise and smoking, really. So, I had to do the right thing; get rid of the worst one first.”* *(Male interviewee, age 55)*

*“I think whoever is managing the site needs to make some decisions about some goals which are going to be fairly universal… I think then you could negotiate out some others if you wish with your GP, and your GP can say to you “Well here’s the list of goals” …and the ones that are important to you, go away and enter those.”* *(Male focus group participant, age 63)*

*“To think of having, say, two or two-and-a-half cups of vegetables for a not very big eater, you know, I wouldn't be eating anything else*. *That didn't really get to me.” (Male interviewee, age 84)*

#### 2. Persuasive category: Dialogue support

Category principles such as praise, reminders, rewards and suggestion aided the users’ primary task. For example, recognition of achievement with virtual rewards for goal tracking influenced personal motivation and self-esteem in terms of healthier lifestyle behaviour. Participants described that goal setting focussed their efforts, establishing a personal challenge and the determination to reach goals. Missed achievement prompted a sense of disappointment but greater effort. Achievement generated positive feedback loops, for example, walking a longer distance or doing so more frequently.

*“I was fairly lazy about following a nutrition program, and also fairly lazy about making sure I got the right amount of exercise. So the big change is that every day it was a reminder to tick the boxes and see what I’ve achieved through the day.”* *(Male interviewee, age 65)*

*“Every smoker wants to give up smoking. But when you start registering your achievements or your goals every time, it's like ‘yep, gone another day, gone another day’.”* *(Male interviewee, age 55)*

Category principles of reminders, suggestions and praise were contained in semi-personalized lifestyle- and medication-related messages received by email and/or short message service (SMS). Of the survey respondents who reported using the email format (n = 322), 55% found the emails helpful. Of those who reported using SMS format (n = 267), 54% found the messages helped them. Analytic data indicated that for participants who opted into message receipt over the 12 months of study follow-up, on average participants received SMS for 9.5 months (SD 4.10) and emails for 10.6 months (SD 3.10). Participants could vary their own uptake of this feature at any time. Those who opted in to this feature described many advantages as well as potential improvements. Feedback indicated that the messages both supported the users’ primary tasks and enhanced the overall persuasive effect. For example, regular message receipt focused attention on one’s health more globally, not only on the specific task or suggestion within a single message. One participant commented that a message might be a reminder to eat fruit today but the effect was that she thought more carefully about general grocery purchases or meal planning.

#### Language, frequency and variety affected receptiveness to the message content

Recipients appeared to place high value on quality, interesting, informative, thought-provoking content conveyed in simple language; overly simplistic content tended to be construed as patronising and to detract from the message/recommendation. Some perceived that high message frequency diluted their impact and regularity was boring; others found the pre-set frequency acceptable. Message receipt at random times of the day is preferable to set times because the unpredictability helps maintain interest. There was strong preference for variety and less repetition of messages, as repetition diminishes impact; for others, repeating message content was acceptable.

*“I like the fact that it was at different times because I know myself if it was the same time every day I would not have looked at it.”* *(Female focus group participant, age 68)*

*“I felt the information was just often terribly basic*. *So, I would think less frequent, and more considered. (Male focus group participant, age 82)*

#### Healthier behaviour was positively influenced when message content was delivered repeatedly and perceived to be practical

Most recipients described the message benefit in terms of prompting adoption or modification of behaviour(s) around healthier eating, healthier food purchases, physical activity, and medication adherence. Importantly, participants described weighing up whether the suggestions were feasible in their particular circumstances and then tried to adopt them as routine. Regularity of message receipt was an advantage for many in the way that it put the change/action ideas on their mind for some period prior to them actually adopting the new behaviour. For others, sporadic desirable behaviours became more habitual, along with anticipation of improving health by taking up the suggestions/recommendations.

*“It was just that, planting that seed in the back of my head that yes, I've got to do that. And being constantly told that you’ve got to do something, I think*, *it's like your mother isn’t it? I don’t know, you tend to go and do it.” (Female interviewee, age 51)*

*“Sometimes I just delete, but they kept coming. It was at you all the time, and in order to make changes in your lifestyle, you really need that. Eventually it does*, *you do make the change and it then becomes part of your life, like exercise now, for example.” (Male focus group participant, age 68)*

*“I don't have salt anymore*. *I get those little messages…all the different things that you can do, and I think, ‘I'll try that’.” (Female interviewee, age 66)*

#### Message receipt benefits new and existing heart health awareness

Participants reported that message content was often information they already knew, but most found that acceptable, considering it was good to be reminded, or to have existing knowledge reinforced. Similarly, message content provided reassurance that some existing habits were among those recommended for heart health. For others, receiving familiar information was sufficiently irritating that they opted out of receiving messages. Overall, message receipt benefitted new and existing heart health awareness with value placed on informative and educational content, with personalisation.

*“I do have a program, like my own fitness program, based on the information I’ve had from this and the reminders that I get from this. I’ll go and do my walking, think, ‘Oh I don’t feel like it’ but I walk anyway. I used to like celery and the fruit and all those types of things*. *I’d eat a piece every now and then, but because of this, it’s become more of a habit, a good habit.” (Male interviewee, age 77)*

*“I didn’t mind them because, yeah, I’m doing that anyway. So, it was just reassurance*. *‘Okay, you’re doing the right thing’…which is nice.” (Male focus group participant, age 72)*

#### 3. Persuasive category: System credibility support

Participants generally found guideline-derived CVD prevention content and in particular, the EHR-derived content, to be trustworthy information. Health and personal data security concerns were mentioned by patients for future integrated health record initiatives but not as a reason to reject the idea in principle. A long-standing relationship with the GP furthers the sense of trust in the health information uploaded or accessed by that provider. National peak bodies with a well-regarded reputation for information were considered trustworthy as partners in digital health resources, and local rather than overseas web sites were felt to be more credible for medical information. Generally, consumers disapproved of commercial entities profiting from or marketing with personal health data. Patients feared unlawful access and/or access by third parties who might use data to the consumer’s disadvantage, for example for targeted advertising, or to adversely affect private health insurance premiums and benefits. Others were more concerned about the consequences of non-health data breaches, for example of bank account data.

*“I think disadvantages would be if somebody can access information illegally to say well*, *so and so needs a beta blocker and I’ll just target him with…emails and SMS.” (Male focus group participant, age 61)*

*“…I would look at partnering with people like the National Heart Foundation and numerous other heart research groups around the country.”* *(Male focus group participant, age 67)*

Reading from credible sources of medication information, for example, facilitated information-seeking about prescriptions and conversations with the prescriber. Participants liked being able to learn about their medications in terms of side effects, impact on other conditions they have, and pertinent questions for the prescriber or pharmacist. Another reported benefit of seeing a current, updateable prescriptions list from their EHR was not having to remember medication names and dosages when asked by a provider other than their GP. Participants were receptive to content of heart health message tips that mirrored familiar information from other sources.

#### Verifiability and authority appeared to positively influence risk awareness and, in turn, health behaviours

*“15% of us will be dead in a few years. I thought right, I’ve got to change that…it got through to me, that’s what bad shape I was in*. *It sort of sunk in.” (Male focus group participant, age 73)*

*“But this was in your own house and you’re online and nobody is going to say, ‘You’re stupid,’ because you just click a little button and the information comes up and you go, ‘Oh, so I’m taking that for that reason’ Now I’ll go and have a look at what other things can fix up high cholesterol*. *So, I’d do a bit of research and a bit of personal reading. And the next time I went to the endocrinologist I had some questions already from reading about things.” (Female interviewee, age 57)*

*“I…started to ask the chemist all about that…I was reading my medications…that were actually on the screen. I was interested in my medications and wanted to know exactly…what the long-term effects and that were of the medications. So now, when the doctor says I'll just give you this, I say, yeah but, is that going to affect my liver, or is that going to affect my kidneys and all that. Whereas before, I would have just took it*. *I'm much aware. I actually ask the doctor, too, now.” (Female interviewee, age 58)*

*“Me and my wife, we just sat down and had a chat about it and just changed the mix of what’s on the plate, the right proportions and stuff like that, eat less, eat a better mix*. *Almost, we’ve eliminated sugar, salt has gone down…and they seem to be paying off according to my cardiologist.” (Male focus group participant, age 69)*

#### Health literacy influenced perceived credibility of their CVD risk score estimation

Although the score was personalized, patients spoke of it seeming less relevant in isolation from other risk factors (e.g., family history), or an investigative test (for example, negative coronary angiogram) that is not incorporated into the score estimation; or when a contributing measurement to the score estimation was perceived by the patient to be erroneous (for example, a one-off high blood pressure reading at an office visit).

*“It was clear that mine was … driven by my genetics, my family history*. *It seemed to me that the dial was not going to change anything.” (Male interviewee, age 63)*

*“According to the study my heart health wasn’t very good, but I know my heart health is very good so that tends to make the believability a lot less*. *And that’s been proven to me because of the [normal] angiogram.” (Male interviewee, age 63)*

#### 4. Persuasive category: Social support

Eleven percent (n = 44) of respondents reported using the chat forum/message board. For those who did engage with this feature, it was used to read messages written by others (61%), read messages by the study team (52%); share personal experiences (34%); and ask questions (18%). Two different themes described user reaction to this feature. First, social and chat forums lacked relevance and appeal for those disinterested in social media. Participants without a more general interest or participation in social media platforms found this feature of low appeal. Some felt their age, or their personality, was not suited to the medium and they preferred not to interact with strangers:

*“I was more interested in my conversations with the doctor and myself*, *and my own records, rather than sharing my information with everybody else.” (Male focus group participant, age 62)*

*“It’s not me*. *It’s not my personality type.” (Male interviewee, age 55)*

Second, sharing common experiences could be helpful to those who are comfortable with using an anonymous chat forum. More patients held a casual interest in viewing the screen if they were otherwise logged into their portal but preferred interesting health-related content over other people’s comments about, for example, their holidays. Even if they were not personally interested, participants agreed that the feature could serve as a support for those wishing to know about others who have a similar condition or problem:

*“There were…some good things on there. Like people were saying that they'd cut out fatty foods and they exercise more*. *But I didn't actually put that on there. Maybe I should have. Maybe give someone else a bit of incentive.” (Male interviewee, age 64)*

*“Some people need to know that someone else has done it.”* *(Female interviewee, age 78)*

Interestingly, it appeared that social support was felt by some participants who had opted into receiving healthy lifestyle and medication tips. Beyond the actual message content, regular messages receipt conveyed a more general sense of interest and concern from the study team about the recipient’s well-being. Messages were felt by some recipients to represent human contact, even though the message delivery was automated and one-way:

*“You haven’t been forgotten*. *I suppose somebody my age, that reads these things, sometimes you tend to think they’re personalised, although they’re not, you know. So that’s what I meant by saying it keeps people, sort of, “Oh, CONNECT’s just got in touch! That was the way I saw it.” (Male interviewee, age 69)*

*“It was an outside contact*, *which I don't have much of…It was like somebody else cares.” (Female interviewee, age 66)*

*“I just felt not alone if you know what I mean, that someone was caring that I was going to be all right*. *I just felt so sure of being looked after and knowing what to do.” (Female interviewee, age 78)*

### GP perspectives on the integrated eHealth intervention and future similar initiatives

GPs mostly identified advantages to their patients’ EHR being integrated with the consumer-focused portal (Table 4). Efficient data transfer to the patients’ portal had potential to improve health care quality; and promoted greater patient involvement with their care and enhanced communication with their provider. A minority of GPs commented on time required to manage software issues related to the record integration. Few data security concerns were raised for this particular application; however this issue was commonly raised by GPs when they spoke about the wider use of record integration between consumers and providers. Further, the fact of patients viewing their own data when it was uploaded by the GP was felt to be more beneficial for patients that a static or less personalised web site. Themes related to how the GPs perceived the effect on their patients who used the EHR-integrated portal were (1) that CVD knowledge and personal risk awareness improved; (2) EHR-linkage improved health-related behaviour and attitude; (3) EHR-linkage facilitated a positive experience of the web application.

*“If there was no new information to add, then I would think, ‘Oh, you know, I’d better check their blood pressure, I’d better check their weight.’ It just sort of put a bit of onus on me to make sure there was something each time I saw them*, *which helped me as well if I overlooked to do their weight, to check their blood pressure.” (M, Interviewee #4)*

*“They were being proactive about their own check-ups, rather than waiting for something to happen*. *One said, ‘can you check my blood pressure today because I’m in the CONNECT study’… it’s increasing their engagement.” (M, Interviewee #15)*

*“They felt it was worthwhile, of interest, that they’ve got motivated, increased motivation to be diligent in contributing to the data and to follow up appointments*. *I didn’t know how to predict what would happen, so it’s been positive.” (M, Interviewee #13)*

**Table 4:** GP experiences and views of an EHR-integrated, consumer-focused portal.

**1. Perceived advantages and disadvantages of the patients’ EHR being integrated with a consumer portal**	
Theme 1: Efficient data transfer from the EHR to the patients’ portal has a quality improvement benefit; confirmation to the provider is helpful	*“The biggest advantage was that it saved a lot of time because all the information was extracted automatically so we…didn’t have to collate anything*, *we didn’t have to look for the information*.*” (*M, Interviewee #4)
	*“The software was easy*, *you just had to click a button and upload everything*, *so that was very simple*.*”* (F, Interviewee #8)
	*“It kind of felt like a lot of stuff was just happening behind the scenes…It was minimally intrusive*.*”* *(*M, Interviewee #1)
	*“If there was no new information to add*, *then I would think*, *‘Oh*, *you know*, *I’d better check their blood pressure*, *I’d better check their weight*.*’ It just sort of put a bit of onus on me to make sure there was something each time I saw them*, *which helped me as well if I overlooked to do their weight*, *to check their blood pressure*.*”* (M, Interviewee #4)
	*“I wish it had been more linked in with our clinical care*.*”* (Survey respondent)
Theme 2: Integration offers greater patient involvement with their health care	***“****Patient can easily access medical information about themselves*, *promotes patient autonomy*.*”* (Survey respondent)
	*“More patient involvement in their healthcare”*. *(*Survey respondent)
	*“I think there were certainly some benefits…to the patients who’d been actively involved and setting those goals and trying to target them*, *being encouraged and getting accountability*. *And if that’s improved their health outcomes by reducing risk*, *we all win*.*”* (M, Interviewee #13)
	*“They were being proactive about their own check-ups*, *rather than waiting for something to happen*. *One said*, *‘can you check my blood pressure today because I’m in the CONNECT study’… it’s increasing their engagement*.*” (*M, Interviewee #15)
	*“The people that I’m recognising as having gone through this program are making more of an effort than those that haven’t*.*” (*F, Interviewee #17)
	*“We do see patients coming back more*, *some of them*, *so I think that’s good*. *I think you’ve achieved what you’re supposed to achieve*.*” (*M, Interviewee #7)
Theme 3: Integration enhances communication between provider and patient	*“[Patients] felt more connected and supported with health goals*.*” (*Survey respondent)
	*” Overall their management became more systematic*.*” (*Survey respondent)
	*“In principle*, *I like the idea of that sort of thing*. *It’s the conversation that it promotes as much as anything*.*”* (M, Interviewee #9)
	*“I’ve always been open to the patients having access to results and co-sharing in the decision-making*. *So*, *that’s been re-affirmed*.*” (*M, Interviewee #14)
	*“The most useful feedback I had was actually discussing what the patients got back from it and sort of*, *‘Have you seen it*? *Have you done anything as a result*? *Do you need interpretation*? *Do you need to discuss anything*?*’ It was sort of that linkage was there*.*” (*M, Interviewee #6)
	*“I am aware of what the patient can see and factor that in to any discussion/consultation*.*”* (Survey respondent)
Theme 4: Record integration and software management requires provider time	*“There were a couple of hiccups on the software…interference with some of our factors that had to be adjusted and dealt with but it was dealt with well*.*” (*M, Interviewee #13*)*
	*“Time issues in general practice and time needed to familiarise self*.*” (*Survey respondent)
	*“In day to day practice I am so busy I really don't need another thing to monitor*!*”* (Survey respondent)
**2. Impressions of the effects on their patients who used the EHR-integrated portal**	
Theme 1: CVD knowledge and personal risk awareness improved	*“More awareness about risks of CVD*.*” (*Survey respondent)
	*“Better understanding*.*” (*Survey respondent)
	*“Realise the situation*.*” (*Survey respondent)
	*“I think it’s a good thing even just even in terms of bringing up awareness because they’re all you know to a degree preventable risk factors*.*” (*M, Interviewee #12)
Theme 2: EHR linkage improved health-related behaviour of patients.	*“More regular attendance*.*”* (Survey respondent)
	*“Self-monitoring of their health + health goals*.*” (*Survey respondent)
	*“Improved diet*, *improved exercise*.*” (*Survey respondent)
	*”I guess they were being proactive about their own check-ups*, *rather than waiting for something to happen*. *Yes*, *it’s increasing their engagement” (*M, Interviewee #15)
	*“They would say*, *‘Yes*, *we want to check on this’*, *or ‘I’ve seen my last results and I’d like to get help’*, *or “I understand that this result implies this and I’d like to improve it’ or something of that kind*.*”* *(*M, Interviewee #6)
Theme 3: EHR linkage modified attitude of patients towards their health care	*“Increased motivation for behaviour change*.*” (*Survey respondent)
	*“Patient feeling in control with access to their data*.*”* (Survey respondent)
	*“Patient feeling more confident in self-management*.*” (*Survey respondent)
	*“It engages the patient to give them some responsibility too*, *some ownership of their problems*. *It takes some pressure off us too because they log things in and they take part of the responsibility and…they can then visually see the progress that they’re making and I think that’s a big motivating factor*.*” (*M, Interviewee #4)
	*“They felt it was worthwhile*, *of interest*, *that they’ve got motivated*, *increased motivation to be diligent in contributing to the data and to follow up appointments*. *I didn’t know how to predict what would happen*, *so it’s been positive*.*” (*M, Interviewee #13)
Theme 4: EHR linkage facilitated positive experience of the web application	*“Novelty aspect*.*”* (Survey respondent)
	*“Greater access to their results of investigations without an appointment needed*.*” (*Survey respondent)
	*“I remember one person saying*, *‘Oh*, *how come that [pathology result] is not there yet*?*’ You know*, *they were looking for it*. *They were keen to see it come up*.*” (*M, Interviewee #3)
	*“Other patients loved the fact that they can access the results and so on*, *and sometimes they get the results before they came to see me because it would upload sooner and they’d see those results*.*” (*M, Interviewee #14)

Abbreviations: CONNECT, Consumer Navigation of Electronic Cardiovascular Tools; CVD, cardiovascular disease; EHR, electronic health record.

When surveyed about characteristics of patients that would make them likely to recommend to patients a technology-based tool for CVD prevention, the dominant considerations were comfort with technology (44.3%, 27/38); younger age (24.6%, 15/38); and willingness to use devices for health (21.3%, 13/38). Conversely, factors that would make GPs less likely to recommend such an intervention included: disinterest or low technical skills (38%, 21/38); elderly age (29%, 16/38); and unwillingness to use devices for this purpose (23.6%, 13/38). Interestingly, socioeconomic status, education level, literacy and cognitive deficit were rarely mentioned as influential characteristics. One GP interviewee commented:

*“The key thing is not to presume*. *Because by presuming something you might not try something in a patient which might actually work for them…people you might think are unlikely to succeed with a digital tool may surprise you.”*

Views of GPs about the wider role of integrated portals to support patients and patient care mirrored in part those of consumers (Table 5). GPs were concerned about the potential risk to health data security of portals linked between consumer and provider; however, they were unanimously in favour of technology-based approaches supporting greater and more active health care involvement by their patients.

*“I think you’ve got to be extremely technology savvy to make sure that your other information is protected*, *and to try and figure out who the trustworthy sources are…I don’t want to be the leaking source.” (F, Interviewee #2)*

**Table 5:** GP views about the role for integrated portal approaches for patients and providers.

**Theme 1: Evidence of effectiveness and long-term use is important**
• *“I think that what [the study] is doing is working at an important level as opposed to a glitzy level*.*”* (M, Interviewee #3)
• *“It might remind them about their diet* .* *.* *. *for three or four or five or six months*, *and then after that the novelty will wear off*.*”(M*, *Interviewee #5)*
• *“Most of the stuff I’ve seen is quite useless*. *Consumer-focussed things tend to be apps focussed on the number of steps you do a day or tracking your weight… and they’re not really giving you the full story* .* *.* *. *unless they can be integrated and actually produce something useful*.*” (M*, *Interviewee #14)*
• *“I just think the whole idea is a very positive one and potentially a very useful one*, *but the question will be whether it works*.*”(*M, Interviewee #15)
• “*If there’s evidence to support its use*, *then you should*.*”(*M, Interviewee #1).
**Theme 2: Potential to enhance patient engagement, but caution about reduced interpersonal counsel from providers**
• *“There’s an approach that says all we need to develop is apps and that’s our problem solved*. *I worry that it can be seen as a sort of modern solution that’s going to solve all our woes…it takes people out of the equation*.*” (*M, Interviewee #9)
• *“I think with a lot of those things–lifestyle change…maybe we’ll spend a lot of time in one consult but at subsequent consults we’re looking at other things…we don’t sort of reinforce it and then the patient*, *they might usually think ‘Well*, *it’s not that important*.*’ So I guess with eHealth the big advantage is that…even if it’s just automated messages…then they know they’ve got someone who’s constantly checking*. *When you know that someone’s going to be checking on things you stay committed*, *you stay focused*.*”* (M, Interviewee #4)
• *“I see people who use those ‘Fitbits’ as pedometers and things like that*. *It gives them motivation because then they do extra incidental activity*, *so I guess eHealth is a big area and it CAN assist patients*, *but they also have to be willing to make those changes*, *so there’s a difference*.*” (*F, Interviewee #8)
• *“I think people having access to it* .* *.* *.*in a way where they can easily refer back [to GP] and not get totally spooked*.*” (*M, Interviewee #6)
**Theme 3: Alternative resource formats are always necessary**
• *“What suits one doesn’t suit another*. *‘What’s the best way to deliver the information*?*’ Any way*. *As many ways as possible*.*”(*F, Interviewee #16)
• *“Anything is useful*. *Whether it be a hard copy or technology*. *If they’re pensioners and what have you*, *often they won’t have relatively good technology…barrier would be the cost*.*”* (F, Interviewee #2)
• *“I have patients who don’t know how to work their phone*. *They can make it do a phone call…they don’t really know how to use the internet particularly*.*” (*M, Interviewee #9)
• *“I could imagine that my relatively well-educated Anglo background patients might well do much better with this than say non- English speaking background people in [nearby suburban area name]*.*” (*M, Interviewee #15)
• *”Half of my patients have mental health issues…they’re not very satisfied with dealing with electronic stuff*.*”* (F, Interviewee #11)
**Theme 4: Potential to improve convenience and accessibility of decision-support resources for providers**
• “It makes good sense for there to be a master portal…for the [Australian Medical Association’s] Doctor Portal [to be] integrated more into Best Practice*. *It’s probably not that hard to click on Google and log into Doctor Portal and stuff but it’s nice if things can be all logged in at the same time*.*” (*M, Interviewee #3)
• “I think we are more interested in properly incorporating…a lot of the simple calculated things into our software that my Medical Director* *can use*. *I don’t want to open up a million web sites just to punch the number in because our computer may not be fast enough to load web sites*, *but if we can incorporate it in the future in some kind of software*, *I think that will be easier for us*.*”* (M, Interviewee #7)
• *“One of the messages that keeps on coming through is the importance of embedding the recommendations from the national guide [lines] into clinical software*. *Because that's actually what influences clinicians' behaviour*. *Availability of recommendations… at one end of the spectrum*, *and the other is like actually drawing information from the record to make recommendations in real time and do decision support*. *Especially with the degree of comorbidity we have…what are the other conditions that can play into this*? *I think the computer doesn't keep up with actually the patients that we're seeing*.*”* (M, Interviewee #9)
• *“GP work gets more complex all the time*. *So we appreciate any way we can work the workflow a little bit better in our decision-making*. *So there’s great opportunity but it’s unfulfilled at the moment*.*” (*M, Interviewee #14)
**Theme 5: Potential risk to health data security**
• *“I think you’ve got to be extremely technology savvy to make sure that your other information is protected*, *and to try and figure out who the trustworthy sources are…I don’t want to be the leaking source*.*” (*F, Interviewee #2)
• *“One always needs to be cautious about the confidentiality*.*”* (M, Interviewee #15)
• *“Potential for software conflict/data mismatch*.*” (*Survey respondent)
• *“Possible 'hacking' and breach of confidentiality*.*” (*Survey respondent)
**Theme 6: Thoughtful, tailored design is important**
• *“Being very specific*. *That’s what patients like*, *when you really go into those specifics*. *Concrete*. *So they can set long-term goals*. *I think that’s sort of where eHealth can come in*.*” (*F, Interviewee #8)
• *“The biofeedback side of things is likely to be helpful as long as it’s meaningful*.*” (*M, Interviewee #3)
• *“If it’s clunky and fails and gets in the way and it’s confusing*, *well*, *all that’s a mess*.*”* (M, Interviewee #3)
• *“GPs will complain about time*, *time*, *time*, *time*, *time*, *time…your App*, *your interface*, *needs to be something we don’t have to put too much time into ourselves*.*” (*F, Interviewee #17)
• *“In principle*, *I like the idea of that sort of thing…the risk is that it says that’s all that’s important about your health because that’s what we can measure and show you at the moment*.*”(*M, Interviewee #9)
• *“I think it has to be well thought through and well-targeted*.*” (*M, Interviewee #14)

Abbreviations: CVD, cardiovascular disease; EHR, electronic health record; GP, general practitioner.

*Best Practice and Medical Director are electronic clinical record systems used by many Australian primary health care providers.

Perceptions that a shared record alone is too static could be countered by interactive, health promoting, educational or action-oriented functionality that draws from the consumer’s own data within the portal, in a similar way to the style of intervention evaluated in this study. Whether targeted to the GP or to patients, thoughtful, tailored design of resources is felt to be important. Interestingly, although enhanced patient engagement was viewed as a benefit of technology-based tools, displacing interpersonal health counselling from providers was a caveat mentioned by several GP interviewees, as was the value of GP contributions to design of proposed system-wide innovations. In particular, some interviewees saw the potential to keep improving the relevance and sophistication of decision-support resources for providers, focusing on timesaving and convenience.

*“I think people having access to it …in a way where they can easily refer back [to GP] and not get totally spooked.”* *(M, Interviewee #6)*

*“GPs will complain about time, time, time, time, time, time…your App*, *your interface, needs to be something we don’t have to put too much time into ourselves.” (F, Interviewee #17)*

More caution emerged in the opinions around solely patient-focused technologies, in that evidence of effectiveness and long-term use was felt to be important, but lacking. Alternative resource formats that are not internet-dependent are still valued, for example, where cost, digital literacy and other barriers may limit use by some patients.

*“It might remind them about their diet … for three or four or five or six months, and then after that the novelty will wear off.”* *(M, Interviewee #5)*

*“Anything is useful. Whether it be a hard copy or technology*. *If they’re pensioners and what have you, often they won’t have relatively good technology…barrier would be the cost.” (F, Interviewee #2)*

## Discussion

This evaluation study of a consumer-focused eHealth intervention that is integrated in part with the primary health care EHR identified several persuasive features that are likely to influence its use and potentially improve health-related behaviour. Within the web application, the interactive and personalised functionality was described as having increased CVD risk factor knowledge, motivated healthier behaviours and for some, enhanced patient-GP engagement. These effects were both reported by patients using the intervention and observed by their GPs over the course of the RCT follow-up period. Importantly, participants also identified shortcomings and offered ideas that would have improved the intervention’s personal utility and appeal. The majority of GPs were in favour of a future similar integrated system but identified implementation concerns such as workflow impact, data security, design optimisation, and questions of inequitable consumer access.

### Relationship of included PSD principles to beneficial intervention features

Effective intervention features for facilitating healthier behaviour for patients in this study reflected three user support categories within the persuasive design framework, namely Primary Task support, Dialogue support and System Credibility support. Patients hardly used the chat forum feature of the Social support category, thereby tempering any persuasive effect of the category principles of social comparison or normative influence on adoption of desirable lifestyle behaviours. In this study, one or more characteristics of the participants or the intervention could have accounted for the low appeal of communication with an anonymous online group. Interestingly, automated text- and email messages which by design chiefly reflected principles of the Dialogue support category, were actually described by some recipients as a signal of social support. Similar findings of Primary Task and Dialogue support being prioritised by users over Social support techniques have been reported in studies of development of digital strategies for CVD secondary prevention, [27] and studies of user preferences for features to support physical activity in people with chronic obstructive pulmonary disease.[28] For those with chronic and complex conditions that require substantial engagement with a care provider, more traditional social exchange may therefore be preferable to ‘virtual’ support within a digital device. Conversely, Social support category principles have shown a higher profile in interventions aimed at alcohol and smoking behaviour change [1] A recent systematic review examining persuasive features in web-based eHealth interventions for older adults with chronic physical conditions found that persuasive principles within Primary Task, Dialogue and System Credibility support categories, in that order, were the most utilised persuasive features by the patients, and Social support the least. [29] Similarly, a systematic review of Internet-mediated (but not EHR-integrated) interventions for improving diet, physical activity, weight loss and metabolic risk factors in primary and secondary prevention populations noted that tailored approaches with self-monitoring and goal-setting (Primary Task support) were of greater benefit and uptake than social forums (Social support) [30] Use of at least one technique from Primary Task and Dialogue support categories characterised interventions that positively affected self-care, daily functioning, blood pressure control, lifestyle behaviours and disease knowledge.[29] The eHealth intervention in this study adopted several System Credibility principles, however this PSD category is a noted gap in mobile [31] and web [32] applications despite being highly valued generally by patients using personalised technology-based approaches for self-management. [33] Further research may elucidate what number or specific combinations of techniques from these key PSD categories enable the outcomes seen. It is plausible that a few key principles of each PSD category may work synergistically, or other external factors are important. Multiple specific persuasive principles can be beneficial but additional ones may not necessarily be more helpful. [34]

### Relationship of included PSD principles to triggers and motivators for healthier behaviour

Within a model of human behaviour change proposed as a framework for designing digital resources with a persuasive intent, motivation, ability (or simplicity) and trigger must occur altogether. [35] In this study, for example, viewing biometric and/or pathology and medication data from the EHR appeared to generate triggers and motivators for healthier behaviour. These are instances of Primary Task support, strengthened by the EHR integration (which invokes such principles as expertise, authority, trustworthiness, and verifiability within the System Credibility support category). Participants cited a high absolute CVD risk score, or a poorly controlled vascular risk factor, or the reward of seeing risk factor improvement on a monitoring chart. An adverse risk factor that causes concern or anxiety, contrasted with feeling satisfaction of seeing (or anticipating) improvement, typify the fear/hope motivator pairing in persuasive technologies. [35] Increasingly in digital resources designed for chronic illness, persuasive computing uses self-tracking of biometric data to elicit such an emotional response from the individual as a precursor to behaviour change. [36] Successful triggers gain the user’s attention and are associated with a target behaviour, thereby acting as a reminder to perform that behaviour. [35] Moreover, if the reminder presents an idea that could readily be adopted within daily routine without needing new or more resources, it improves ability to try the target behaviour. When the triad of trigger (reminder), motivation (emotional response to the trigger) and ability occurs the likelihood increases that the target behaviour will happen. [35] Participants in this study described that email and/or SMS heart health message tips containing realistic change/goal suggestions (suggestions are a principle of Dialogue support) improved their ability to adopt a healthier behaviour. Previous studies have noted this to be habit strengthening for desirable dietary and physical activity behaviour in secondary CVD prevention. [37] Participants similarly described how healthier behaviours became more habitual than random, and how goal setting (Primary Task support) and frequent heart health reminders (Dialogue support) aided this effect. Habit formation is believed to facilitate *maintenance* of a behaviour beyond initial uptake and is strengthened by frequent cue exposure.[15, 38]

### Insights for broadening EHR integration

This study provides insights from GPs and patients to inform future development of integrated health records. It makes a timely contribution to the national conversation among consumers, providers and policy-makers about preferences and acceptability for such innovations as the federal government is embarking on an ambitious strategy to establish personal health records for all Australians.[10] Integrating persuasive technologies with personal health records to assist patients with managing their care was identified a decade ago as a challenge yet to be addressed [39] In this study, persuasive principles used in a consumer-facing application with EHR integration was viewed by patients and GPs to positively influence patient engagement and the care relationship. It created expectations from the patient for timely updating and uploading of information by their GP. Patients were enthusiastic about potential expanded interactivity in the system, such as sending back to their GP content entered at home or other information exchange for management decisions. Direct patient-provider communication was not designed into the application used in this study; however, it has been shown to be a valued component for self-care in several chronic conditions [13, 15, 40] Preventive care and health behaviour discussions are seen as pertinent topics for an integrated system to facilitate in keeping with the objective of enhanced provider-consumer communication. [41, 42] An implementation concern cited by GPs for integrated portals requiring content uploads, for example, was further encroachment on time; quantification of this is worth further investigation as previous research suggests this concern is more perceived than real. [11, 18] Further research could also explore models of reimbursement to providers for interacting with patients via the portal. In this study, prescriptions could not be renewed through the portal but seeing their current medication record helped patients identify and correct errors—a safety benefit of patient portals noted in other studies. [11] Interestingly, as stand-alone personal health records evolve to web-based models, a range of health-related applications enable functions like prescription viewing and refilling or laboratory test retrieval. [7, 16] Interoperability of consumer-controlled records with a range of service providers within the health system offers consumers greater accessibility to information and other resources with which to increase health care engagement. Moreover, the potential for interactive options offered by either comprehensive integrated records or tethered health records to advance shared health care decision-making appears to be a target of evolving research in this area. [43, 44] Consideration is warranted for consumers without the skills, confidence, access, or technical and support resources to opt into and effectively navigate these complex systems and could be excluded from experiencing their benefits. [17, 45, 46] In this study, the issue of disparities with technology-enabled healthcare support was a general concern raised by several GP interviewees in respect of some of their own sub-groups of patients; this issue was infrequently mentioned by patients. Another implementation issue concerns the theme of trust in health data security and privacy, which dominates consumer and provider adoption of digital health care innovations. [41, 47, 48] In this study, health data security eclipsed other perceived operational barriers, signalling that wider community trust must be earned by public, corporate, research or other entities seeking to further any national agenda for integrated or tethered health records.

### Study strengths and limitations

Among the strengths of this study is that the evaluation was conducted prior to knowledge of the RCT outcomes by researchers or participants, thereby removing bias from the data collection and interpretation. [49] High survey response rates and mixed research methods were a clear study strength in providing comprehensive information about intervention adoption, experiences and preferences. [50] Identification of PSD categories that were associated with user preferences and benefits builds understanding of the relationship of user experiences with persuasive software features, an under-reported but key interaction for behaviour change support with eHealth. [51]

This study also has several limitations. Recall bias is possible because surveys, focus groups and interviews were conducted after 12 months of study participation. Caution is always advised in interpretation of self-reported impacts of intervention exposure on improving health-related knowledge, attitude and behaviour, although these effects were also observed by GPs. Focus group and interview transcripts were not independently coded, however the emergent themes were regularly discussed, contested and re-interpreted in consultation with the broader study team. There is potential for selection bias as same-day cancellations for focus groups occurred and weekday conduct of focus groups and interviews limited the attendance of some working participants. The study overall recruited a larger majority of males (in part due to the high risk eligibility criteria), but also a disproportionately large number of female patients were unavailable or unwilling to attend evaluation activities. In addition, the mean ages of survey respondents, focus group attendees and interviewees were 66 years, 69 years and 67 years respectively; younger people who have grown up with technology may have different expectations of eHealth strategies for disease prevention and health care navigation and early onset CVD may have several additional implications for this group (such as loss of employment potential). Further research between and within tiers of demographic variation would improve understanding of how these differences influence experience of the persuasive intent of health technologies and expectations of integrated health records.

## Conclusions

Within the wider technology-assisted health space, stand-alone web applications and personal health records are evolving to web-based integration with provider EHRs. Both integrated and tethered models show variable scope, scale and deployment internationally. Using mixed methods inquiry, this study explored the effective persuasive features and ascertained user acceptability of a web application with some EHR integration in a health system context of relatively early experience of such record systems. Furthermore, appraisal of the intervention features and patient experiences against the persuasive design framework noted the relative utility of the four framework categories. These findings aid understanding about digital interventions with a persuasive intent for those with CVD risk factors. We conclude that while there is merit in investing in EHR-integrated consumer portals, more attention is needed to their design and the need to make explicit the persuasive design principles that are at play. This will generate more generalizable knowledge around factors that promote adoption of these portals and assist in the development of future eHealth strategies for behaviour change support.

## Supporting information

S1 File Feedback surveys. (PDF)

S2 File Discussion guides (patient participants). (PDF)

S3 File Interview guide (general practitioner participants). (PDF)
