# Supplementary material for: Persuasive design features within a consumer-focused eHealth intervention integrated with the electronic health record: A mixed methods study of effectiveness and acceptability
Source: PLoS One. 2019 Jun 20;14(6):e0218447. doi: 10.1371/journal.pone.0218447 (PMC6586306; doi:10.1371/journal.pone.0218447)
Supplement: S1 File — (PDF) [file pone.0218447.s001.pdf]

CONsumer **N**avigation of **E**lectronic **C**ardiovascular **T**ools (CONNECT) Study

Supporting Information: End-of-Study Survey (Patient Participants)

1. **Did you have access to the CONNECT Web site in the 12 months you took part in this study?**
○ **NO** → There are no more questions. Please return form to study staff (see instructions at end of survey)
○ **YES** → Please complete the remaining survey questions

PART A - These questions are about using the different features in CONNECT

2. Which types of heart health goals did you set? (select all that apply)

○ Healthy eating	○ Physical activity	○ Wellbeing & mental health	○ Smoking cessation	○ I did not set goals

Please read each statement below and select one response

	**Strongly Agree**	**Agree**	**Neutral**	**Disagree**	**Strongly Disagree**	**Did not use this**
3. Tracking my goals helped me focus on my heart health	○	○	○	○	○	○
4. The Heart Risk dial helped me understand how changing risk factors would affect my overall risk	○	○	○	○	○	○
5. The email message tips helped me	○	○	○	○	○	○
6. The text message tips helped me	○	○	○	○	○	○
7. It was *not* helpful to add my personal measurements to graphs (e.g., for blood pressure)	○	○	○	○	○	○
8. The 'More info' buttons and links to other Web sites were *not* useful	○	○	○	○	○	○

9. How did you use the "Join the Chat" message board?
○ I did not use this → Please go to Part B   ○ I read and/or wrote comments → Please go to Q.10

10. What did you like about the "Join the Chat" message board? (Select all that apply)
○ sharing my experiences ○ asking questions ○ reading messages written by others
○ reading messages from the study team ○ other____

PART B - These questions are about taking part in CONNECT

11. On average, how often did you log in to use CONNECT?
○ Never ○ Less than once each month ○ A few times each month ○ At least once each week

12. It was hard for me to find the screens and features I needed ○ Yes ○ No

13. Information on the screens was clear to me ○ Yes ○ No

14. Did phone and email access to the study staff help you? ○ Yes ○ No
If yes, please give 1 or 2 examples:

15. For me, using the CONNECT program for 12 months was
○ A suitable duration ○ Too long ○ Too short

PART C - These questions are about how taking part in CONNECT affected you				
16. I was motivated to improve my heart health because (select all that apply)				
○ My blood cholesterol level was high	○ My heart risk score was high	○ My blood pressure or weight was high	○ My GP's advice	○ I have a family history of heart disease
17. As a result of CONNECT, I am better at taking my prescribed medicines				
○ Strongly agree	○ Agree	○ Neutral	○ Disagree	○ Strongly disagree
18. As a result of CONNECT, I do more to improve my mental health and well being				
○ Strongly agree	○ Agree	○ Neutral	○ Disagree	○ Strongly disagree
19. As a result of CONNECT, my weekly physical activity level is				
○ Much higher than before	○ A little higher	○ About the same	○ Less than before	
20. As a result of CONNECT, my eating habits are				
○ Much healthier	○ A little healthier	○ About the same	○ Less healthy	
**PART D - Please provide any further feedback about the following questions**				
21. Which screens or features did you find most useful? Why? Did you have a favourite?				
22. Which screens or features did you find least useful? Why?				
23. How could we improve the CONNECT program?				

CONsumer **N**avigation of **E**lectronic **C**ardiovascular **T**ools (CONNECT) Study

Supporting Information: End-of-Study Survey (General Practitioner Participants)

The RecordConnect software installed on your computer enabled linkage of the practice or clinic electronic record with the patient's personalized CONNECT Web site.

24. What concerns, if any, did you have about the software for this purpose?

    ____

    ____

25. Were your concerns addressed/resolved by CONNECT study staff? ○ Yes ○ No
    Comment: ____

	Strongly Agree	Agree	Neutral	Disagree	Strongly Disagree
26. Overall, I found it easy to use the RecordConnect software for conduct of this trial	○	○	○	○	○
27. I am satisfied with the support from the CONNECT study staff during the study start-up and recruitment period at my practice or health service	○	○	○	○	○
28. During the periods of initial participant recruitment and follow-up study visits, the presence of CONNECT staff was disruptive to my practice or health service	○	○	○	○	○

29. For what reasons did you agree to take part in the CONNECT trial? (Select all that apply)
    - □ The concept of eHealth for patient care interests me
    - □ I already comfortably use eHealth systems in my practice/clinic
    - □ I could see potential benefits for my patients who took part
    - □ Taking part required minimal time input from me/my staff
    - □ The aims of the study aligned with my personal research interests
    - □ The option to claim CPD points for taking part appealed to me
    - □ Other: ____

30. The CONNECT Web site was innovative in that it was linked to your patient's medical record in your practice or health service software. What do you feel are the benefits and/or drawbacks of using an integrated system such as this?

    ____

    ____

    ____

31. Would you be in favour of a similar linked program in the future? ○ Yes ○ No
    Comment: ____

32. The CONNECT Web site included a risk dial that displayed for the patient their personalized absolute CVD risk score. Do you use the absolute risk score to explain CVD risk to patients and/or to initiate conversation about lifestyle behaviour change? ○ Yes ○ No
    Comment: ____

33. For patients at moderate-high CVD risk, which strategies do you use for lifestyle behaviour counselling or advice? (Select all that apply)
    - □ Printed materials
    - □ Face-to-face discussion
    - □ Desk-based software tools - e.g. HealthTracker CVD risk projection graph
    - □ Referral to other health professionals - e.g. dietitian, hospital-based cardiac rehab (if applicable)
    - □ I suggest use of commercial programs/devices – e.g. "Weight Watchers", activity monitoring devices
    - □ I suggest use of technology ("Apps") for supporting behaviour change – e.g. "My QuitBuddy"
    - □ I suggest community-based activities/resources – e.g. local walking group, gym, pool
    - □ I request that the patient track or record measurements in between office visits – e.g. BP, weight
    - □ Other: ______________________________

34. How likely are you to recommend to your patients an eHealth strategy for lifestyle behaviour change?
    O Very likely   O Likely   O Unlikely   O Very unlikely

35. Which patient characteristics make you *likely* to recommend a technology-based tool for CVD prevention?
    ____
    Why? ______________________________

36. Which patient characteristics make you *unlikely* to recommend a technology-based tool for CVD prevention?
    ____
    Why? ______________________________

37. Did any of your patients taking part in CONNECT initiate conversation with you about their use of the program?   O Yes   O No
    If yes, what was discussed? (e.g., goals set, receipt of motivational tips, improved activity levels, etc.)
    ____

38. What were three beneficial features or aspects of the CONNECT program for your patients who took part?
    a. ______________________________
    b. ______________________________
    c. ______________________________
    Comment: ______________________________

39. Were you aware of any improvements in laboratory or physical measurements for your patients who used the CONNECT program (e.g. BP, weight, BMI, LDL-C, HDL-C, HbA1c)?   O Yes   O No
    Comment: ______________________________

40. How often do you participate in research?   O Never   OSometimes   O Often   O Very Often

41. How often do you conduct your own research?   O Never   OSometimes   O Often   O Very Often

42. Is there anything else you'd like to add regarding the CONNECT trial?
