# Supplementary material for: Persuasive design features within a consumer-focused eHealth intervention integrated with the electronic health record: A mixed methods study of effectiveness and acceptability
Source: PLoS One. 2019 Jun 20;14(6):e0218447. doi: 10.1371/journal.pone.0218447 (PMC6586306; doi:10.1371/journal.pone.0218447)
Supplement: S2 File — (PDF) [file pone.0218447.s002.pdf]

<u>**CON**sumer **N**avigation of **E**lectronic **C**ardiovascular **T**ools (CONNECT) Study</u>

<u>Supporting Information: Focus Group Discussion Guide</u>

1. Overall, what did you think of the CONNECT heart health program?
2. What were some of the things you liked?
3. What do you think about goal setting for lifestyle habits that you wanted to improve?
4. Your CONNECT Web site was linked in with your GP's medical records. How relevant or important is that for you?
5. How useful is it to see and chart measurements of your BP or weight, for example?
6. How did you use the Heart Risk screen with the dial that showed your risk for a heart event in the next 5 years?
7. Did you read and/or add comments to the "Join the Chat" message board?
8. Information was provided about heart conditions, tests and medicines – how useful were the 'More info" buttons or the Web site links?
9. What did you think about the healthy tip messages that you could choose to receive by email or by SMS?
10. What features in the CONNECT program helped you think about risk for heart disease or want to make some lifestyle changes?
11. What types of things stop you or help you with changing lifestyle habits?
12. How do you feel generally about using technology to help with healthy lifestyle?
13. Was there a key thing within CONNECT that influenced you to improve healthy behaviour?
14. What other healthy lifestyle programs or activities did you use at the same time as you were using CONNECT?
15. How easy or difficult was it to find things and use different features?

16. The study staff contacted you regularly and were available by phone and email at all other times. What do you think of the support provided by the study staff?
17. Did you discuss being part of a research study with your family, friends or doctors? What did they think about the program?
18. Invite final comments.

CONsumer **N**avigation of **E**lectronic **C**ardiovascular **T**ools (CONNECT) Study

Supporting Information: Interview Guide (Patient Participants)

1. Could you tell me about your experience with improving your heart health during the time you were taking part in the CONNECT research study?
2. How do you feel about using technology in everyday life to help with improving health?
3. In what ways do you think technology can be helpful for people who are trying to increase healthy lifestyle behaviour; what features do you feel are important?
4. Which type of people might be likely to use technology to help them increase healthy behaviour?
5. For you, what role does technology have or could it have in helping you with healthy lifestyle?

There seems to be many different things that affect whether people can make healthier changes in daily life, for example to eat more fruit and vegetables, or do more physical activity in their day.

6. For you, what stops you or makes it harder to do the healthy things you'd like to do?
7. For you, what makes it easier to do the healthier lifestyle things you want to do?

People are motivated to improve their heart health for many different reasons.

8. For you, what were reasons to start or increase healthier behaviour? Is there anything that acted as a trigger for you?
9. Can you tell me about things in the CONNECT program that you read or used that helped you think about risk for heart disease, or made you want to make some lifestyle changes?

Programs that use technology or the Internet to help people with healthier lifestyle often have a few specific features. I'd like to ask you about these in relation to how you used the CONNECT program.

10. Your CONNECT Web site was linked in with your GP's medical records. How important for you is seeing your blood results or measurements compared with recommended targets?
11. What do you think about goal setting for lifestyle habits that you wanted to change or improve?
12. For you, how useful is it to share your story or progress with others on a social media or 'Chat forum".

Thinking about other interactive parts of the program:

13. How useful did you find the heart risk dial with the sliding bars that showed how risk changes when your measurements change?
14. What did you think about the healthy tip messages that you could receive by email or by SMS? What effect did they have?
15. How does charting your measurements or seeing updated results of blood pressure or weight, for example, help you stay on track?
16. How has using the CONNECT program changed your understanding about heart health risks and ways to lower the risk?

From talking to other people, I have learned that sometimes people set goals for one particular area of lifestyle, such as healthier eating or getting more physical activity; others try to make changes in more than one area.

17. Can you tell me about areas of your lifestyle where you made changes or improvements?

18. Could you describe what are you doing differently now compared to before you took part in CONNECT?
19. What do you think made it possible for you to make the changes you have made?
20. What aspects of your lifestyle do you feel are now better or are about the same as before?
21. Is there anything else you would like to tell me that I haven't asked you about?
