# Supplementary material for: Persuasive design features within a consumer-focused eHealth intervention integrated with the electronic health record: A mixed methods study of effectiveness and acceptability
Source: PLoS One. 2019 Jun 20;14(6):e0218447. doi: 10.1371/journal.pone.0218447 (PMC6586306; doi:10.1371/journal.pone.0218447)
Supplement: S3 File — (PDF) [file pone.0218447.s003.pdf]

CONsumer **N**avigation of **E**lectronic **C**ardiovascular **T**ools (CONNECT) Study

Supporting Information: Interview Guide (General Practitioner Participants)

1. What experience have you had personally, or has your practice had, with eHealth activities?

For the conduct of the CONNECT trial, you will recall that we installed software called RecordConnect on your computer to enable linkage of the patient's electronic health record with their personalised CONNECT Web site.

2. What advantages and drawbacks do you see in a consumer-focussed program that is linked in this way?
3. If a similar linked program was available again in the future (outside a clinical trial setting), can you tell me about reasons you would or would not use it?
4. Did any of your patients who were using CONNECT mention it to you during their office visits?
5. If so, could you give me some examples of comments (positive or negative) they made?

The CONNECT Web site showed patients a risk dial that displayed their absolute CVD risk score and they could move interactive sliders to see how adjusting the value of drivers of that score changed their risk.

6. Can you tell me about how you use the absolute risk score to explain CVD risk to patients and/or to initiate a conversation about lifestyle behaviour change?
7. Could you tell me about some of the other ways that conversations start with patients about lifestyle behaviour change? Initiated by GP or by patient?
8. How easy or difficult is it for patients to understand combined risk factor status versus a single factor as the driver of absolute risk for a CVD event?

9. What are some of the strategies you use for mod-high CVD risk patients to engage them in healthier lifestyle behaviour?
10. Could you describe the patient characteristics that affect your choice of strategy(ies)?
11. Thinking about the reasons why patients find it hard to initiate or maintain behaviours to improve risk factors, in what ways do you think an eHealth strategy could help support and sustain behaviour change?
12. For which type of patient would you be likely to recommend a technology- based tool for CVD prevention or would you expect to see succeed with one? Why?
13. For which patients would you be unlikely to recommend such an approach? Why?
14. Prior to taking part in CONNECT how did you feel about the role of eHealth approaches to CVD risk reduction in primary health care?
15. How has taking part in the CONNECT trial changed any of your views?
16. Is there anything else you'd like to tell me that I haven't asked you about?
